# Examining the Psychometric Properties of Four Mattering Constructs Through Rasch Modeling: Evidence From Turkish Adolescents and College Students

**DOI:** 10.1002/brb3.71634

**Published:** 2026-07-28

**Authors:** Alican Kaya, Zane Asher Green, Mehmet Şata, Murat Yıldırım, Abdulselami Sarıgül, Nuri Türk, Mirko Duradoni

**Affiliations:** ^1^ Department of Guidance and Psychological Counselling Ağrı İbrahim Çeçen University Ağrı Türkiye; ^2^ Psychology Research Center Khazar University Baku Azerbaijan; ^3^ Contemporary Research Initiative Preston University Islamabad Pakistan; ^4^ Department of Educational Sciences, Faculty of Education Van Yüzüncü Yıl University Van Türkiye; ^5^ Department of Psychology Faculty of Science and Letters Ağrı İbrahim Çeçen University Ağrı Türkiye; ^6^ Department of Sociology Faculty of Science and Letters Ağrı İbrahim Çeçen University Ağrı Türkiye; ^7^ Department of Guidance and Psychological Counselling Faculty of Education Siirt University Siirt Türkiye; ^8^ Department of Education, Literatures, Intercultural Studies, Languages and Psychology University of Florence Florence Italy

**Keywords:** adolescents, anti‐mattering, college students, mattering, online anti‐mattering, online mattering, psychometrics

## Abstract

**Background:**

Mattering and anti‐mattering are central constructs in understanding individual well‐being. Traditionally, these concepts have been studied in offline social contexts. The rise of information and communication technologies has expanded the social environments in which individuals may feel valued or, conversely, insignificant. However, evidence on measurement invariance of these combined offline and online mattering scales for gender and different developmental stages remains limited in culturally diverse countries, including Turkey.

**Objectives:**

This study aimed to validate four scales assessing both offline and online dimensions of mattering and anti‐mattering—the General Mattering Scale, the Anti‐Mattering Scale, the Online Mattering Scale, and the Online Anti‐Mattering Scale—within a Turkish sample of adolescents and college students.

**Methods:**

Data were collected from 888 participants (high school students: *n* = 434, mean age = 15.51 ± 1.02, 63.1% female; college students: *n* = 454, mean age = 21.10 ± 1.67, 84.1% female). Confirmatory factor analyses (CFA) were conducted to examine the dimensional structure and reliability of the scales. Additionally, the Many‐Facet Rasch Model (MFRM) was applied to test measurement invariance across educational levels and gender.

**Results:**

CFA results indicated acceptable fit indices for all four scales across both groups, with reliability coefficients exceeding recommended thresholds. MFRM analyses further supported measurement invariance across high school and college students, as well as across male and female participants.

**Conclusion:**

The four‐scale set appears psychometrically sound for use with Turkish adolescents and emerging adults and is well‐suited to capturing the complexity of contemporary hybrid social environments, where experiences of significance and neglect can occur both offline and online.

## Graphical abstract

This study examined the psychometric properties of four mattering constructs—general mattering, anti‐mattering, online mattering, and online anti‐mattering—among Turkish adolescents and college students. Using data from 888 participants, the researchers applied confirmatory factor analyses to test the dimensional structure and reliability of the scales, and employed the Many‐Facet Rasch Model to assess measurement invariance across gender and educational levels. Results showed that all four scales demonstrated acceptable fit indices and strong reliability, with invariance confirmed across both high school and college samples as well as male and female participants. These findings suggest that the scales are robust tools for capturing how young people experience significance and neglect in both offline and online social contexts, providing valuable insight into well‐being in contemporary hybrid environments.

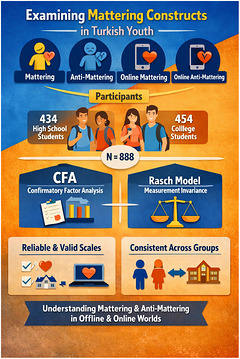

## 1 Introduction

The belief that one is important to others fulfills a basic psychological need. This awareness is critical to the progress of emotional, social, and mental health across time (Rosenberg and McCullough 1981; Flett 2018). As such, mattering plays a significant role in psychological research. The sense of importance, esteem, significance, and being truly valued and appreciated by others are the ingredients that make up mattering. Conversely, the sense of being overlooked, ignored, unnoticed, or unimportant in social settings is the core of anti‐mattering (Flett et al. 2022). It has been shown that perceived mattering has a positive correlation with happiness, social connectedness, and psychological resilience. On the contrary, anti‐mattering is associated with loneliness, emotional distress, and depressive symptoms (Demir et al. 2012; Paradisi et al. 2024; Tonini et al. 2025). Mattering has become a notable topic in recent years, especially among adolescents and young adults who are in the developmental stages marked by high sensitivity to social judgments, social group identification, and identity construction (Arnett 2000). Based on the developmental framework of Arnett (2000), in this study, high school students are conceptualized as adolescents and college students as emerging adults. The perception of social importance is especially relevant to psychological adaptation and well‐being during these stages, when adolescents and young adults are mainly eager to be liked by peers, family, and other important people (Schlossberg 1989; Shafiq et al. 2023). It has been shown that instabilities or impairment in the sense of mattering during these developmental stages can have long‐term implications on emotional well‐being and quality of interpersonal relations, and that these effects will spill over into later developmental stages (Flett et al. 2022).

Meanwhile, modern social spaces are becoming more and more digital and online in nature as opposed to face‐to‐face interactions alone. In these settings, the experiences of mattering as well as the feelings of exclusion or invisibility among youth groups can be intensified (Odgers and Jensen 2020). A recent study has also found that mattering in both offline and online environments is positively associated with digital well‐being. On the other hand, anti‐mattering is associated with higher levels of problematic social media use (Duradoni et al. 2024). These findings suggest that feeling socially valued in both in‐person and digital contexts may support healthier online engagement, whereas feeling overlooked may contribute to maladaptive use of social media.

Online and face‐to‐face interactions have several differences. In digital environments, social approval is often made explicit through indicators such as comments, likes, and follower counts. In online spaces, however, there tend to be fewer nonverbal cues and communication may not always occur in real time. Such characteristics of online spaces can influence how individuals make sense of acceptance, attention, and exclusion in social situations. As such, online mattering and anti‐mattering experiences may not fully capture the essence of in‐person interactions (Duradoni et al. 2024; Odgers and Jensen 2020). This distinction suggests the importance of studying both constructs separately as well as considering how they may complement each other.

Realizing the importance of mattering experiences and to effectively assess them, there exist several validated measures. The most prominent of these are the General Mattering Scale (GMS‐6) and the Anti‐Mattering Scale (Flett and Nepon 2024; Flett et al. 2022). Additionally, previous research has examined these measures for their psychometric properties. That work provides evidence on their factor structure, reliability, and related constructs in non‐Western cultural settings (e.g., Ding et al. 2025; Chen, 2007). Despite these efforts, a comprehensive psychometric analysis still needs to be conducted that takes into account factorial validity, good model fit, and measurement invariance across groups. This stringent investigation is likely to ensure that the observed differences reflect actual or meaningful differences in psychological experiences and not just be the result of measurement issues introduced on account of scale translation or cultural interpretation of items (Hambleton 1994; Van de Vijver and Leung 1997).

Relevant to mention here is that although much emphasis has been placed on the constructs of mattering, the psychometric measurement of these constructs still has gaps. Most of the literature that exists is found on Western samples, which restricts cross‐cultural generalization (Demir et al. 2012; Ding et al. 2025). Also, few studies have examined mattering and anti‐mattering simultaneously and evaluated their psychometric consistency across participant groups (Flett et al. 2022; Ding et al. 2025; Van de Vijver and Leung 1997; Chen 2007). As such, the present scholarly contribution considers the psychometric soundness of mattering and anti‐mattering measures in Turkish high‐school and college student samples with respect to factor structure, reliability, and measurement invariance across age and gender groups.

### 1.1 Significance and Rationale of the Study

The justification and importance of the present study is based on three major considerations. First, there is a pressing necessity for stringent psychometric validation of the measures related to mattering in various cultural settings, following the principles of test adaptation and testing (Hambleton 1994; International Test Commission 2017). In the absence of this evidence, the generalizability and applicability of these instruments are restricted to the contexts in which they were originally developed. This is especially relevant in the Turkish context. Turkey reflects a blend of both collectivistic and individualistic influences. Essentially, these cultural influences shape how people understand their relationships, sense of belonging, and social significance. As a result, mattering and anti‐mattering may be experienced differently from what is normally observed in predominantly Western settings. Examining these scales in the current context demonstrates their broad utility. This contribution therefore contributes to strengthening the cross‐cultural applicability of mattering‐related constructs.

Second, existing methodological studies highlight the significance of assessing structural validity, model fit, and reliability through a combination of multiple complementary indices as opposed to utilizing a single index (Hu and Bentler 1999; Linacre 2002a). An integrated psychometric approach increases trust in measurement quality and makes empirical results more meaningful (MacCallum et al. 1996; Kline 2011). This scholarly contribution is particularly important, as it examines factor structure, reliability, and measurement invariance across important demographic groups. Invariance is a fundamental step in deciding whether differences observed are the result of true psychological differences or bias caused by the process of measurement (Van de Vijver and Leung 1997; Chen 2007). The invariance testing in mattering‐related measures has been conducted prior to this study (e.g., Ding et al. 2025). However, there is limited evidence for combined offline and online scales in a single framework as well as for different developmental stages such as high school and college students in culturally diverse contexts outside Western settings. This study addresses this gap by examining offline and online dimensions concurrently within Turkish high school and college students.

Third, understanding mattering and anti‐mattering in specific developmental and social contexts has become even more relevant with the change in the pattern of interactions between people, such as the growing importance of digital communication (Odgers and Jensen 2020). The presence of reliable and consistent measurement provides the basis for future studies that are required to examine the relationship between these experiences and well‐being, adjustment, and mental health over time and across circumstances (Duradoni et al. 2024).

In addition, by evaluating mattering and anti‐mattering together, the research acknowledges that feeling valued and insignificant are closely related but distinct psychological experiences with different well‐being and distress implications (Flett et al. 2022; Nepon and Flett 2024). This dual focus makes it possible to better comprehend social significance, especially in the stages of development that can be described as the most vulnerable to social exclusion and interpersonal pressure (Arnett 2000; Shafiq et al. 2023).

Thus, this study examines the psychometric properties of four mattering‐related scales in Turkish samples, namely, the GMS‐6, Anti‐Mattering Scale, Online Mattering Scale, and Online Anti‐Mattering Scale. The focus is on factorial validity, reliability, and measurement invariance for gender as well as high school and college students.

## 2 Method

### 2.1 Sample and Design

The study sample consisted of 888 participants. Data were collected simultaneously from high school (*n* = 434) and college (*n* = 454) students using a convenience sample. Before data collection, a regression‐based power analysis was conducted only to obtain a preliminary estimate of the minimum sample size (*n* = 234). However, because the main analyses of the present study were confirmatory factor analyses (CFA) and Many‐Facet Rasch Model (MFRM), sample size adequacy was evaluated primarily according to the methodological requirements of these approaches. In CFA, sample size adequacy depends on model complexity, the number of estimated parameters, factor loadings, and overall model fit rather than a single regression‐based effect size (MacCallum et al. 1996; Wolf et al. 2013). In MFRM, sample size adequacy is related to the number of observations across facets such as persons, items/tasks, raters, and rating‐scale categories; stable estimates generally require sufficient observations for each facet element and response category (Linacre 1994; Linacre 2002b). Therefore, the final sample sizes for both the high school and college groups were considered adequate for the CFA and MFRM analyses conducted in this study. While 63.1% (274) of high school students are female and 39.9% (160) are male, when looking at their class levels, 36.9% (160) are in ninth grade, 34.6% (150) are in 10th grade, and 28.6% (124) are in 11th grade. When their socioeconomic levels were examined according to their self‐assessment, 12.4% (54) stated that they were at a low level, 78.6% (341) at a medium level, and 9.0% (39) at a high level, and it was determined that the average age of these individuals was 15.51 ± 1.02. While 84.1% (382) of college students are female and 15.9% (72) are male, when looking at their class levels, 51.5% (234) are in the 1st year, 14.8% (67) are in the 2nd year, and 33.7% (153) are in the 3rd year. When their socioeconomic levels are examined according to their self‐assessment, 18.3% (83) stated that they are at a low level, 77.3% (351) at a medium level, and 4.4% (20) at a high level, and it was determined that the average age of these individuals is 21.10 ± 1.67.

### 2.2 Measures

#### 2.2.1 GMS‐6

The GMS‐6 was first developed by Rosenberg and McCullough (1981). Flett et al. (2022) subsequently developed a five‐item version of the scale. Finally, Flett and Nepon (2024) added one more item to the scale, resulting in a six‐item version. The items on the scale (e.g., “How important are you to others?”) are rated on a four‐point Likert scale from 1 (not at all) to 4 (a lot). The GMS‐6 consists of six items and a single dimension. Scores on the scale range from 6 to 24. It is assumed that mattering increases with higher scores. The internal consistency coefficient of the scale was found to be 0.87 in the study where it was developed.

### 2.3 Anti‐Mattering Scale

The Anti‐Mattering Scale was used to assess participants' levels of anti‐mattering (Flett et al. 2022). The items on the scale (e.g., “How much do you feel like you don't matter?”) are rated on a four‐point Likert scale from 1 (not at all) to 4 (a lot). The Anti‐Mattering Scale consists of five items and a single dimension. Scores on the scale range from 5 to 20. It is assumed that anti‐mattering increases with higher scores. The internal consistency coefficient of the scale was found to be 0.86 in the study where it was developed.

### 2.4 Online Mattering Scale

The Online Mattering Scale was used to assess participants' online mattering levels (Duradoni et al. 2024). The scale items (e.g., “How important are you to others online?”) are rated on a four‐point Likert scale from 1 (not at all) to 4 (a lot). The Online Mattering Scale consists of five items and a single dimension. Scores on the scale range from 5 to 20. It is assumed that higher scores indicate higher online mattering. The internal consistency coefficient of the scale was found to be 0.81 in the study where it was developed.

### 2.5 Online Anti‐Mattering Scale

The Online Anti‐Mattering Scale was used to assess participants' levels of online anti‐mattering (Duradoni et al. 2024). The scale items (e.g., “How much do you feel like you don't matter online?”) are rated on a four‐point Likert scale from 1 (not at all) to 4 (a lot). The Online Anti‐Mattering Scale consists of four items and a single dimension. Scores on the scale range from 4 to 16. It is assumed that higher scores indicate higher online anti‐mattering. The internal consistency coefficient of the scale was found to be 0.81 in the study where it was developed.

### 2.6 Translation Process

In the process of adapting the scales for Turkish high school and college students, the scale adaptation guidelines in the “International Testing Commission (ITC) Guideline: The Process of Adapting a Psychological Measurement Instrument” were followed (International Test Commission 2017). Accordingly, the necessity of these scales for our country was first determined, and then the necessary permissions for adaptation were obtained from the developers of the original scales. In the adaptation process, the items, response options, and instructions of the scales, which were originally in English, were first translated into Turkish by two researchers. After reviewing the translation form, it was necessary to test whether the items in the original form were equivalent to the items in the translated form. To this end, the items translated into Turkish were back‐translated into English by two language experts. Subsequently, an expert evaluation form was prepared containing both the original instructions, items, and response options of the scales, along with their translations. This form was evaluated separately by an expert group of eight (two language experts, five psychological counseling and guidance experts, and one measurement and evaluation expert) consisting of field and language specialists. As a result of the evaluation, the most agreed‐upon translation for each item was incorporated into the scales by the field and language experts. The final forms were then administered to the participants in the study sample.

### 2.7 Data Analysis

In data analysis, descriptive statistics were first performed to determine the sociodemographic variables of the participants. Then, to examine the psychometric properties of the measurements obtained from the Turkish adapted measurement instruments, Cronbach's *α* and McDonald's *ω* coefficients were used for reliability analyses, and CFA and multifaceted Rasch analysis (MFRM) were used for validity analyses. Before proceeding with CFA analyses, the necessary assumptions were tested. First, it was examined whether there was any missing data in the dataset, and it was determined that there was no missing data. Considering the impact of outliers, the presence of outliers was examined, and it was determined that there were no outliers. To test the assumption of multivariate normal distribution of the dataset, a Mardia test was performed, and it was determined that the data did not exhibit a multivariate normal distribution. For the high school sample, the distributions showed notable deviations from normality. Mattering was highly skewed (skewness = 3.61, *p* < 0.001) with extreme kurtosis (62.71, *p* < 0.001). Anti‐mattering also displayed skewness (1.99, *p* < 0.001) and high kurtosis (40.44, *p* < 0.001). Online mattering was moderately skewed (1.26, *p* < 0.001) with elevated kurtosis (42.21, *p* < 0.001), while online anti‐mattering was strongly skewed (4.24, *p* < 0.001) with kurtosis of 35.01 (*p* < 0.001). For the college sample, similar patterns emerged. Mattering was highly skewed (5.64, *p* < 0.001) with kurtosis of 66.18 (*p* < 0.001). Anti‐mattering showed skewness of 2.40 (*p* < 0.001) and kurtosis of 45.17 (*p* < 0.001). Online mattering was closer to normal (skewness = 0.95, *p* < 0.001) but still had high kurtosis (45.49, *p* < 0.001). Online anti‐mattering was skewed (3.95, *p* < 0.001) with kurtosis of 38.51 (*p* < 0.001). Since multivariate normality was not achieved, the diagonally weighted least squares (DWLS) method was preferred as the parameter estimation method in CFA. CFA analyses were performed using the Mplus (version 8) statistical package program. Inter‐item pairwise correlations were examined, and it was determined that there were no correlations greater than 0.80; in other words, there was no multicollinearity problem, and linear relationships were found between the variables.

CFA was conducted to evaluate the hypothesized factor structure of the scale. However, CFA does not directly model person measures, item difficulty, and additional facets such as group and gender effects within a single measurement framework. Therefore, MFRM was used as a complementary psychometric method. MFRM extends the basic Rasch model by incorporating multiple facets beyond persons and items and allows person, item, and facet effects to be estimated simultaneously (Linacre 1989; Engelhard 2013). In this study, MFRM was preferred over separate differentially functioning items (DIF) procedures because it provided a unified framework for examining item functioning across group and gender facets. Accordingly, CFA provided evidence for factorial validity, whereas MFRM provided additional evidence for item functioning, measurement fairness, and the stability of person and item estimates across relevant facets.

## 3 Results

The study first presents descriptive statistics for both the high school and college samples.

Descriptive statistics showed that the mean mattering score was 2.90 (SD = 0.60) in the high school sample and 3.03 (SD = 0.56) in the college sample. Anti‐mattering scores were slightly higher among high school students (*M* = 2.22, SD = 0.75) than among college students (*M* = 2.08, SD = 0.68). Online mattering scores were also higher in the high school sample (*M* = 2.56, SD = 0.72) than in the college sample (*M* = 2.50, SD = 0.72). Online anti‐mattering had the lowest mean scores in both samples, with similar values for high school students (*M* = 1.81) and college students (*M* = 1.80). The descriptive statistics for the study variables are presented in Table 1. For both high school and college samples, CFA was used to provide evidence of the validity of the measurements obtained from the measurement instruments, and Cronbach's alpha and McDonald's omega coefficients were used for reliability evidence. The findings are presented in Table 2.

**TABLE 1:** Descriptive statistics for high school and college samples.

Scale	High school (*N* = 434)				College (*N* = 454)			
	Mean	SD	Skew.	Kurt.	Mean	SD	Skew.	Kurt.
Mattering	2.90	0.60	−0.35	−0.34	3.03	0.56	−0.37	−0.08
Anti‐mattering	2.22	0.75	0.25	−0.53	2.08	0.68	0.47	0.01
Online mattering	2.56	0.72	−0.09	−0.37	2.50	0.72	−0.02	−0.31
Online anti‐mattering	1.81	0.64	0.43	−0.58	1.80	0.64	0.35	−0.72

**TABLE 2:** Model fit statistics and reliability values.

	High school				College			
Measures of fit	Mattering	Anti‐mattering	Online mattering	Online anti‐mattering	Mattering	Anti‐mattering	Online mattering	Online anti‐mattering
*χ* ^2^/df	1.492	2.142	2.476	2.298	2.002	1.334	0.948	1.445
RMSEA	0.034	0.051	0.058	0.055	0.047	0.027	0.000	0.031
RMSEA of 90% CI	0.000–0.069	0.000–0.094	0.017–0.100	0.000–0.122	0.012–0.078	0.000–0.075	0.000–0.064	0.000–0.104
CFI	0.995	0.993	0.992	0.995	0.991	0.998	1.000	0.999
NNFI/TLI	0.991	0.986	0.984	0.984	0.985	0.996	1.000	0.996
SRMR	0.020	0.019	0.016	0.015	0.020	0.037	0.030	0.028
Cronbach *α*	0.821	0.841	0.855	0.794	0.841	0.850	0.872	0.833
McDonald *ω*	0.827	0.844	0.855	0.797	0.849	0.853	0.874	0.836

Table 2 shows that the tested models have acceptable fit values ​​for both the high school and college samples. The *χ* ^2^/df statistic for all tested models ranged from 0.948 to 2.476, which is considered acceptable (MacCallum et al. 1996). Similarly, the RMSEA values ​​ranged from 0.000 to 0.058, which is also considered acceptable (MacCallum et al. 1996). It was determined that CFI values ​​ranged from 0.992 to 1.000, NNFI/TLI values ​​ranged from 0.984 to 1.000, and SRMR values ​​ranged from 0.015 to 0.037, indicating a good fit (Hu and Bentler 1999).

The path coefficients for the DFA models tested for the high school sample appear to be acceptable. For the high school sample, the standardized path coefficients (factor loadings) were found to be in the range of *λ* = 0.38–0.82, while the error variances were in the range of *ε* = 0.34–0.86. Factor loadings of 0.30 and above indicate that the items are suitable for measuring latent structure, and error variances below 0.90 indicate an acceptable error amount in measuring latent structure (Kline 2011).

**FIGURE 1:**
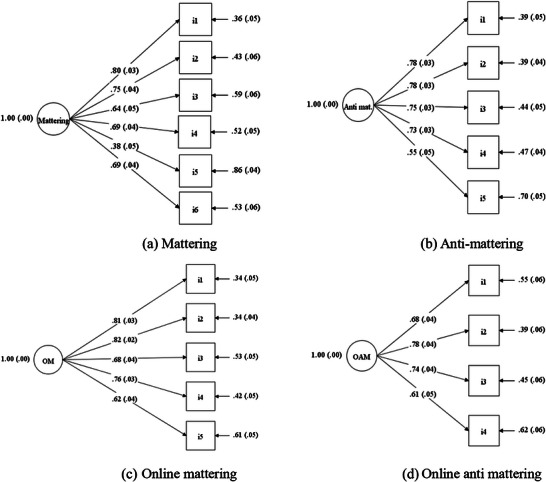
Path diagrams of measurement instruments for the high school sample. Abbreviations: OAM, online anti‐mattering; OM, online mattering.

**FIGURE 2:**
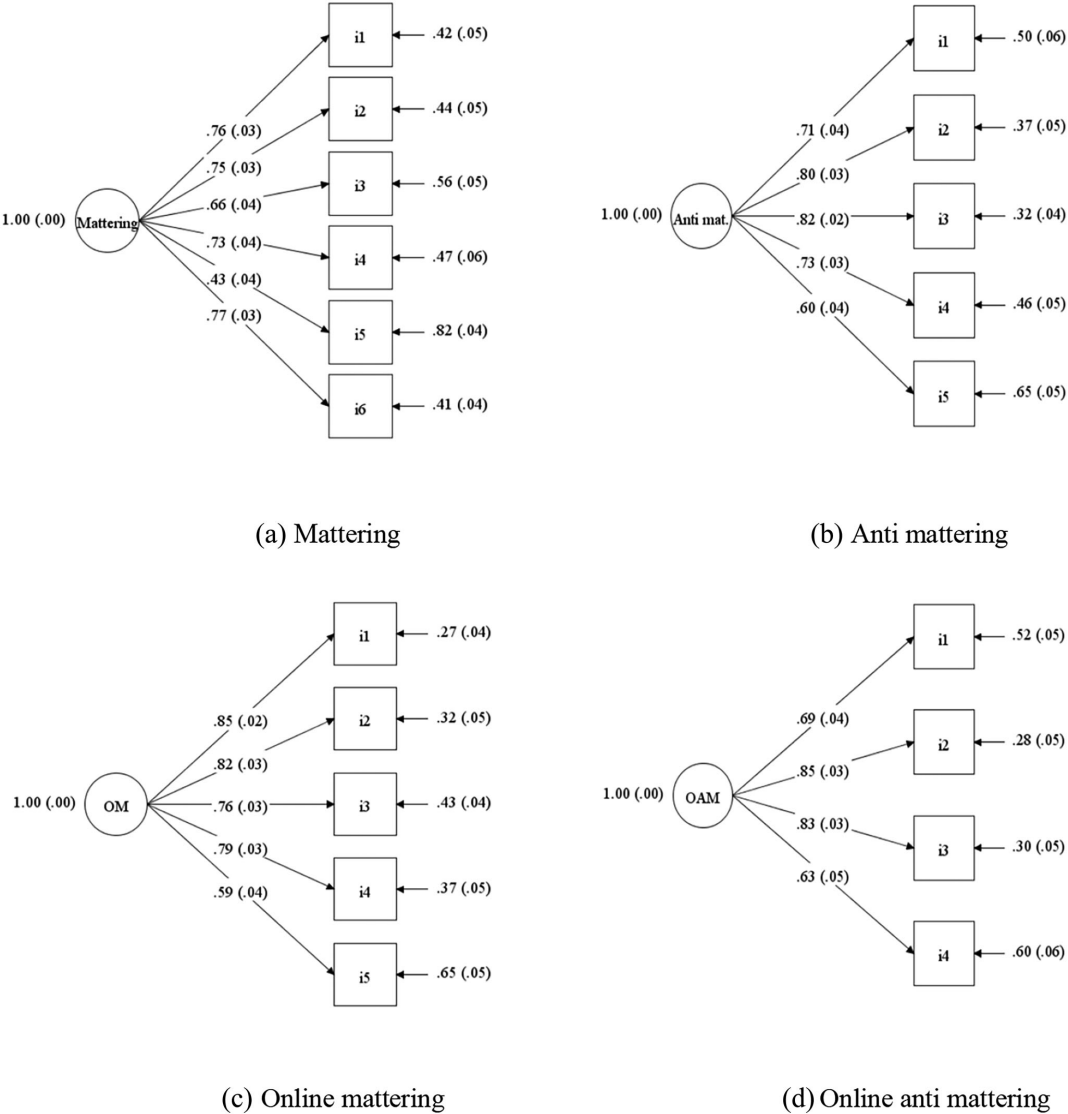
Path diagrams of measurement instruments for the college sample. Abbreviations: OAM, online anti‐mattering; OM, online mattering.

The path coefficients for the DFA models tested for the college sample appear to be acceptable. For the college sample, the standardized path coefficients (factor loadings) were found to be in the range of *λ* = 0.43–0.85, while the error variances were in the range of *ε* = 0.27–0.82. Figures 1 and 2 present the path diagrams of the measurement instruments for the high school and college samples, respectively.

### 3.1 MFRM Analysis

In the Turkish adaptation process of the measurement instruments, after evaluating the psychometric properties, an MFRM analysis was conducted to determine the structural equivalence of the scales in different groups. In this context, it was examined whether the scale represented the same construct for both high school and college samples, and for both male and female individuals. Within the scope of the analysis, item interactions with group variables and potential biases were tested to assess whether the measurement instrument had functional equality at the group level.

**FIGURE 3:**
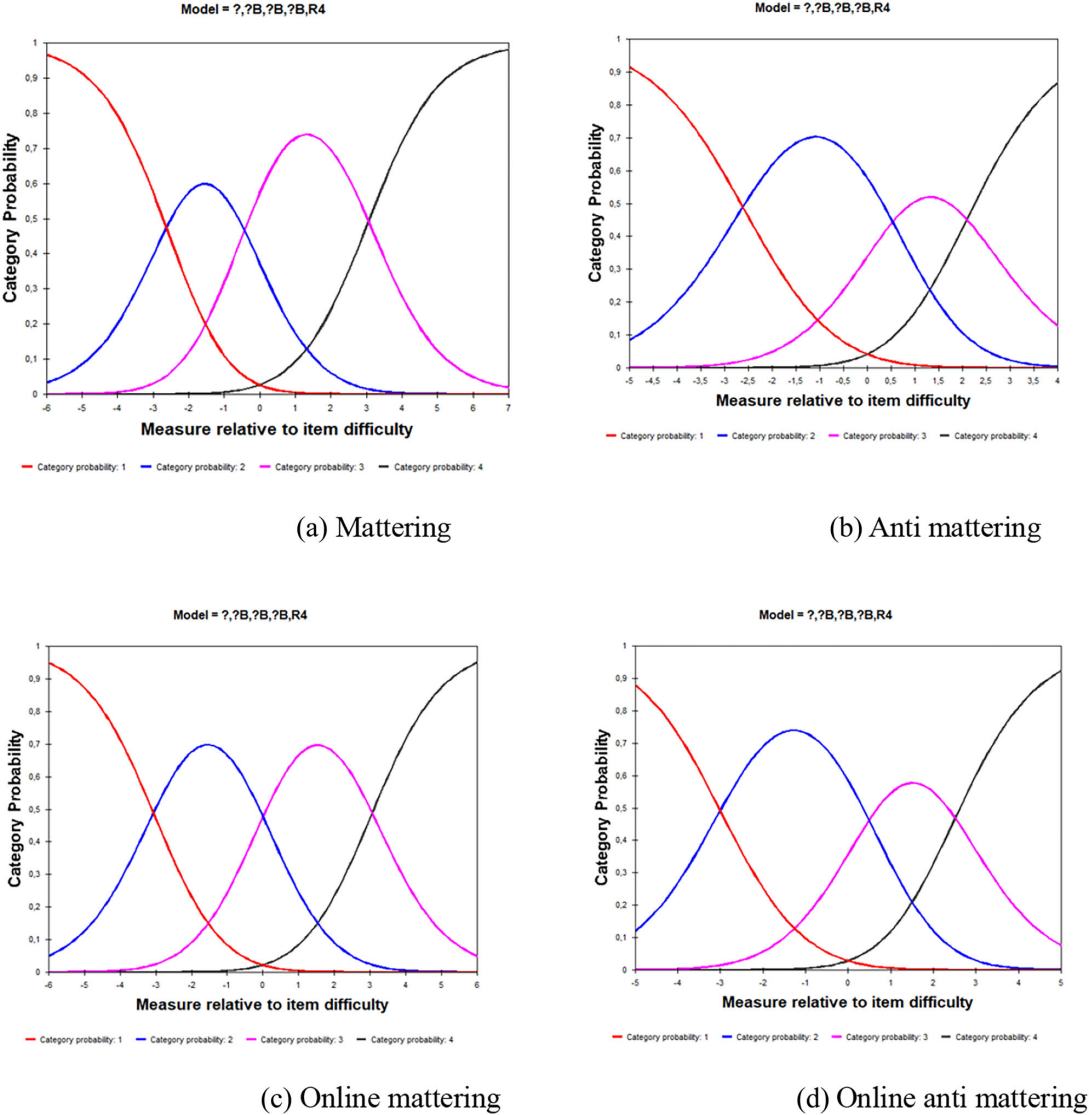
Distribution of item response probabilities for measurement instruments according to measurement dimension.

**FIGURE 4:**
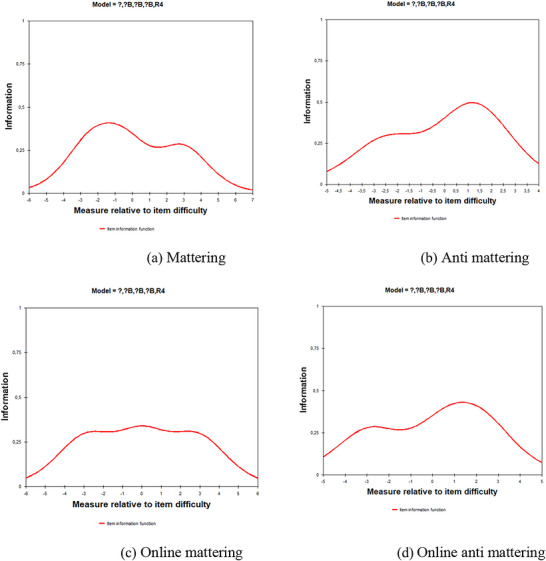
Item information functions related to measurement instruments.

Table 3 presents the results of the MFRM regarding the item‐level interactions of group (high school–college) and gender (female–male) variables for the Mattering, Anti‐Mattering, Online Mattering, and Online Anti‐Mattering Scales. The fixed chi‐square statistic evaluates whether differences among the estimated facet elements are statistically significant after accounting for measurement error. The nonsignificant results indicated that there were no statistically meaningful differences across the modeled facets. Therefore, the findings provided support for measurement invariance and suggested that the scale functioned similarly across the examined groups. The fixed chi‐square (*χ* ^2^) values ​​obtained for all four scales were found to be statistically insignificant (*p* > 0.05). These findings reveal no significant bias or DIF among the items based on either group or gender variables. In other words, the measurement instruments present comparable structures across groups and satisfy the condition of measurement invariance. Figures 3 and 4 present the distributions of item response probabilities across the measurement dimensions and the corresponding item information functions. Also, group x gender x item interactions for the Mattering Scale, Anti‐Mattering Scale, Online Mattering Scale, and Online Anti‐Mattering Scale are presented in Tables 4, 5, 6, 7.

**TABLE 3:** MFRM results regarding group × gender × item interactions for measurement instruments.

Scale	Fixed chi square	df	*p*‐value
Mattering	20.30	24	0.680
Anti‐mattering	18.90	20	0.530
Online mattering	12.10	20	0.910
Online anti‐mattering	8.90	16	0.920

*Note*: In the MFRM framework, the fixed chi‐square statistic tests whether the estimated measures for the modeled facet elements differ significantly beyond measurement error. The degrees of freedom correspond to the number of estimated elements within the relevant facet minus one. Nonsignificant chi‐square values indicate that the modeled facet elements do not show statistically significant variation after accounting for the Rasch measurement model. In the context of this study, nonsignificant *p*‐values suggest that item functioning and scale scores were stable across the relevant facets, providing evidence for measurement invariance across the examined groups.

Abbreviation: MFRM, Many‐Facet Rasch Measurement.

**TABLE 4:** Group × gender × item interactions for the Mattering Scale.

Interaction	Observed score	Expected score	Bias size	SE	*t*‐value	df	*p*‐value	INFIT	OUTFIT
U × M × I1	181.00	182.88	−0.09	0.22	−0.42	65	0.679	0.50	0.50
H × M × I1	439.00	431.70	0.16	0.15	1.06	153	0.289	0.60	0.60
U × F × I1	1005.00	1012.78	−0.07	0.09	−0.74	359	0.461	0.70	0.70
H × F × I1	696.00	693.64	0.03	0.11	0.26	263	0.798	0.80	0.80
U × M × I2	186.00	182.64	0.17	0.22	0.74	65	0.459	0.50	0.50
H × M × I2	431.00	431.17	0.00	0.15	−0.02	153	0.981	0.80	0.80
U × F × I2	996.00	1011.52	−0.14	0.09	−1.47	359	0.143	0.80	0.80
H × F × I2	705.00	692.67	0.15	0.11	1.34	263	0.182	0.90	0.90
U × M × I3	194.00	198.45	−0.23	0.23	−1.03	65	0.308	1.40	1.50
H × M × I3	467.00	468.00	−0.02	0.15	−0.15	153	0.881	1.50	1.50
U × F × I3	1113.00	1098.22	0.15	0.10	1.46	359	0.145	1.10	1.10
H × F × I3	750.00	759.33	−0.12	0.11	−1.05	263	0.297	1.30	1.30
U × M × I4	191.00	185.72	0.27	0.23	1.18	65	0.243	0.70	0.80
H × M × I4	443.00	438.30	0.10	0.15	0.69	153	0.492	1.10	1.00
U × F × I4	1031.00	1028.42	0.02	0.10	0.25	359	0.806	0.80	0.80
H × F × I4	693.00	705.56	−0.15	0.11	−1.37	263	0.172	1.10	1.10
U × M × I5	222.00	227.97	−0.40	0.25	−1.57	65	0.122	1.50	1.40
H × M × I5	531.00	537.06	−0.18	0.17	−1.05	153	0.296	1.60	1.60
U × F × I5	1264.00	1257.74	0.08	0.11	0.71	359	0.477	1.30	1.20
H × F × I5	891.00	885.23	0.09	0.13	0.73	263	0.465	1.70	1.50
U × M × I6	187.00	183.34	0.18	0.22	0.81	65	0.419	0.80	0.80
H × M × I6	428.00	432.77	−0.10	0.15	−0.70	153	0.487	1.10	1.10
U × F × I6	1015.00	1015.32	0.00	0.09	−0.03	359	0.976	0.80	0.80
H × F × I6	697.00	695.57	0.02	0.11	0.16	263	0.877	1.00	1.00

*Note*: Interaction terms indicate combined effects between facets. For example, U × M × I1 refers to the interaction among the university/college group, male, and Item 1.

Abbreviations: F, female; H, high school group; I, item; M, male; U, university/college group.

**TABLE 5:** Group × gender × item interactions for the Anti‐Mattering Scale.

Interaction	Observed score	Expected score	Bias size	SE	*t*‐value	df	*p*‐value	INFIT	OUTFIT
U × M × I1	124.00	118.26	0.29	0.22	1.30	56	0.199	1.50	1.50
H × M × I1	287.00	297.05	−0.21	0.15	−1.44	140	0.153	1.20	1.10
U × F × I1	586.00	572.61	0.15	0.11	1.42	246	0.157	1.10	1.10
H × F × I1	727.00	736.08	−0.08	0.09	−0.83	349	0.406	1.00	0.90
U × M × I2	298.00	301.38	−0.07	0.14	−0.48	142	0.632	1.10	1.10
H × M × I2	110.00	117.89	−0.42	0.24	−1.79	56	0.079	0.50	0.60
U × F × I2	752.00	733.82	0.15	0.09	1.67	349	0.096	0.70	0.70
H × F × I2	564.00	570.91	−0.08	0.11	−0.73	246	0.464	0.70	0.80
U × M × I3	312.00	307.31	0.09	0.14	0.66	142	0.511	1.00	1.00
H × M × I3	121.00	120.21	0.04	0.22	0.18	56	0.860	0.60	0.60
U × F × I3	754.00	747.98	0.05	0.09	0.55	349	0.584	0.80	0.70
H × F × I3	570.00	581.50	−0.13	0.11	−1.21	246	0.226	0.90	0.90
U × M × I4	326.00	316.69	0.18	0.14	1.29	142	0.198	1.20	1.20
H × M × I4	123.00	123.89	−0.04	0.22	−0.20	56	0.844	0.90	0.90
U × F × I4	596.00	598.07	−0.02	0.11	−0.22	246	0.828	1.00	1.00
H × F × I4	764.00	770.35	−0.05	0.09	−0.57	349	0.568	0.90	0.90
U × M × I5	132.00	129.74	0.11	0.22	0.49	56	0.624	0.80	0.80
H × M × I5	331.00	331.57	−0.01	0.14	−0.08	142	0.938	1.30	1.40
U × F × I5	631.00	623.91	0.08	0.10	0.74	246	0.460	1.50	1.50
H × F × I5	797.00	805.78	−0.07	0.09	−0.78	349	0.437	1.20	1.20

*Note*: Interaction terms indicate combined effects between facets. For example, U × M × I1 refers to the interaction among the university/college group, male, and Item 1.

Abbreviations: F, female; H, high school group; I, item; M, male; U, university/college group.

**TABLE 6:** Group × gender × item interactions for the Online Mattering Scale.

Interaction	Observed score	Expected score	Bias size	SE	*t*‐value	df	*p*‐value	INFIT	OUTFIT
U × M × I1	160.00	161.14	−0.06	0.22	−0.26	64	0.799	0.50	0.50
H × M × I1	376.00	377.65	−0.04	0.15	−0.24	147	0.808	0.90	0.90
U × F × I1	861.00	854.40	0.06	0.10	0.64	348	0.524	0.70	0.70
H × F × I1	601.00	604.81	−0.05	0.11	−0.44	249	0.662	0.80	0.80
U × M × I2	172.00	165.38	0.33	0.22	1.48	64	0.143	0.60	0.60
H × M × I2	393.00	387.37	0.12	0.15	0.83	147	0.406	0.90	0.80
U × F × I2	880.00	877.25	0.03	0.10	0.27	348	0.791	0.80	0.80
H × F × I2	606.00	621.01	−0.20	0.11	−1.72	249	0.087	0.80	0.80
U × M × I3	151.00	156.01	−0.25	0.22	−1.12	64	0.265	0.90	0.90
H × M × I3	367.00	365.91	0.02	0.15	0.16	147	0.872	1.20	1.20
U × F × I3	821.00	826.81	−0.05	0.10	−0.56	348	0.575	1.00	1.00
H × F × I3	595.00	585.27	0.13	0.11	1.12	249	0.265	1.50	1.40
U × M × I4	162.00	160.50	0.08	0.22	0.34	64	0.738	0.60	0.60
H × M × I4	378.00	376.18	0.04	0.15	0.27	147	0.788	1.00	1.00
U × F × I4	844.00	850.95	−0.06	0.10	−0.67	348	0.502	0.70	0.70
H × F × I4	606.00	602.37	0.05	0.11	0.42	249	0.677	0.90	0.90
U × M × I5	181.00	182.96	−0.10	0.23	−0.44	64	0.658	1.60	1.60
H × M × I5	421.00	427.89	−0.15	0.15	−1.03	147	0.307	1.30	1.20
U × F × I5	976.00	972.59	0.03	0.10	0.33	348	0.741	1.40	1.40
H × F × I5	694.00	688.54	0.07	0.12	0.63	249	0.531	1.40	1.40

*Note*: Interaction terms indicate combined effects between facets. For example, U × M × I1 refers to the interaction among the university/college group, male, and Item 1.

Abbreviations: F, female; H, high school group; I, item; M, male; U, university/college group.

**TABLE 7:** Group × gender × item interactions for the Online Anti‐Mattering Scale.

Interaction	Observed score	Expected score	Bias size	SE	*t*‐value	df	*p*‐value	INFIT	OUTFIT
U × M × I1	117.00	117.73	−0.04	0.24	−0.18	55	0.861	1.20	1.10
H × M × I1	261.00	260.89	0.00	0.16	0.02	129	0.986	1.10	1.10
U × F × I1	594.00	590.43	0.04	0.11	0.38	285	0.701	1.10	1.00
H × F × I1	431.00	433.96	−0.05	0.13	−0.37	210	0.711	1.20	1.20
U × M × I2	107.00	109.99	−0.19	0.25	−0.75	55	0.459	0.80	0.80
H × M × I2	241.00	243.45	−0.07	0.17	−0.41	129	0.684	0.90	0.90
U × F × I2	551.00	551.39	0.00	0.11	−0.04	285	0.966	0.60	0.60
H × F × I2	411.00	405.18	0.10	0.13	0.75	210	0.452	1.00	0.90
U × M × I3	121.00	117.31	0.21	0.24	0.89	55	0.375	0.90	0.90
H × M × I3	270.00	259.95	0.26	0.16	1.62	129	0.107	0.90	0.90
U × F × I3	577.00	588.34	−0.13	0.11	−1.22	285	0.222	0.70	0.60
H × F × I3	430.00	432.41	−0.04	0.13	−0.30	210	0.763	1.10	1.00
U × M × I4	123.00	122.98	0.00	0.24	0.00	55	0.996	1.20	1.10
H × M × I4	265.00	272.71	−0.20	0.16	−1.22	129	0.225	1.00	1.00
U × F × I4	625.00	616.85	0.09	0.11	0.86	285	0.389	1.20	1.20
H × F × I4	453.00	453.46	−0.01	0.12	−0.06	210	0.955	1.40	1.40

*Note*: Interaction terms indicate combined effects between facets. For example, U × M × I1 refers to the interaction among the university/college group, male, and Item 1.

Abbreviations: F, female; H, high school group; I, item; M, male; U, university/college group.

Findings regarding group × gender × item interactions obtained for each measurement instrument are given in the appendix.

The MFRM analysis performed on the Mattering Scale, Anti‐Mattering Scale, Online Mattering Scale, and Online Anti‐Mattering Scale revealed no significant item‐level bias based on group (high school–college) and gender (female–male) variables. According to the interaction values ​​presented in the table, the *t*‐values ​​for all items were found to be statistically insignificant (*p* > 0.05). This result indicates that the scale maintains its measurement invariance for both high school and college students, as well as for both male and female individuals. The fact that the bias sizes for all items remain below ±0.40 supports the absence of systematic group or gender bias in the scale. Furthermore, the INFIT and OUTFIT values ​​being in the 0.50–1.50 range (Linacre 2002a) indicates that the items fit the model well and that the mismatch between the data and the model is low. Therefore, the Turkish form of the Mattering Scale, Anti‐Mattering Scale, Online Mattering Scale, and Online Anti‐Mattering Scale measures equivalence across both education level and gender groups and is suitable for group‐level comparisons.

## 4 Discussion

### 4.1 Overall Psychometric Performance

The Turkish versions of the GMS‐6, Anti‐Mattering Scale, Online Mattering Scale, and Online Anti‐Mattering Scale have been shown to have strong psychometric properties both at the high school level and at the college level. The predetermined single‐factor structures of all four translated measures were confirmed by CFA. These findings indicate that the items of the scales adequately measure the constructs of mattering and anti‐mattering in offline and online environments (Flett and Nepon 2024; Duradoni et al. 2024). The factor loadings of the scales ranged between adequate and strong, suggesting the appropriateness of the items for measuring the underlying latent constructs. All in all, these results suggest that the Turkish versions of the scales can serve as psychometrically sound measures to assess mattering and anti‐mattering in adolescents and emerging adults (Akyazi and Kavas 2025; Demir et al. 2012). The findings are in line with earlier psychometric research that suggests that mattering‐related scales generally show stable factorial structures and good reliability. Similar results have also been found in Chinese youth samples (Ding et al. 2025).

### 4.2 Patterns of Anti‐Mattering and Mattering

Descriptive patterns indicated higher levels of mattering than anti‐mattering reported by students. This trend is in line with the results of Western and cross‐cultural samples (Ding et al. 2025; Flett and Nepon 2024). Anti‐mattering scores were somewhat greater in high school students compared to college students, and general mattering was marginally higher in the latter than in the former. This indicates developmental differences in perceived social meaning at different educational levels (Arnett 2000; Duradoni et al. 2024). The high school and college student groups reported moderate levels of online mattering. However, high school students reported slightly higher levels of online mattering. Also, the high school and college student groups reported low levels of online anti‐mattering. The aforementioned findings suggest that many Turkish students feel socially valued in online settings, that is, they feel accepted, noticed, and appreciated by others (Duradoni et al. 2024; Shafiq et al. 2023). The patterns of mattering and anti‐mattering indicate minor developmental differences in how Turkish students in both groups perceive being valued or overlooked in their social relationships in both face‐to‐face interactions and digital spaces. Essentially, there is a relatively small difference in both groups’ social experiences in the offline and online domains. These developmental patterns align with prior evidence indicating that social interaction patterns vary from adolescence into early adulthood (Odgers and Jensen 2020).

### 4.3 Factor Structure and Model Fit

The four scales in both samples had acceptable model fit indices (i.e., *χ* ^2^/df, RMSEA, CFI, and TLI) according to the recommended threshold limits (Hu and Bentler 1999; MacCallum et al. 1996). The similarity between the factor structures of the two educational levels (high school and college) adds to the conceptual clarity of the construct validity and stability of mattering and anti‐mattering in the Turkish context. The relatively high model fit indices of online scales suggest that the Turkish youth population of today values digital social experiences and likely feels comfortable in online interactions (Duradoni et al. 2024; Flett et al. 2022). These results imply that the offline and online dimensions of the two constructs are psychometrically distinct and at the same time internally consistent.

### 4.4 Reliability

The internal consistency reliability of the scales, as determined by the Cronbach's alpha and McDonald's omega coefficients, was higher than the recommended cutoffs (Akyazi and Kavas 2025; Flett et al. 2022). Both educational levels had similar reliability estimations, and therefore, the measurement of mattering and anti‐mattering was also similar across high school and college student samples. The findings justify the application of the adapted measures for both research and practical purposes. Thus, these may allow confident interpretation of the scores as well as track changes in mattering and anti‐mattering over time without worrying about measurement errors in the Turkish context (Ding et al. 2025; Duradoni et al. 2024).

### 4.5 Measurement Invariance Between Groups

MFRM analysis demonstrated that across all the scales, no significant item‐level bias was identified with respect to educational level or gender. The measurement invariance was confirmed between the high school–college and male–female groups because the values of biases and the INFIT/OUTFIT measures were within recommended limits (Linacre 2002a; Chen 2007). The findings clarify that the measures may be adequate for making valid comparisons of perceived social significance between developmental stages (high school and college stages) and gender groups.

### 4.6 Developmental and Context Trends

A subtle developmental trend was identified. Compared to high school students, marginally higher general mattering and anti‐mattering were apparent among college students. High school students experienced relatively higher online mattering and slightly higher online anti‐mattering compared to college students. During adolescence and early adulthood, these patterns may reflect age‐related differences with regard to how people develop their sense of identity, evaluate their social worth, and interact with others in both in‐person and digital settings (Arnett 2000; Odgers and Jensen 2020). In view of the findings, considering both face‐to‐face and digital domains together helps in providing a more complete picture of how young people experience mattering and anti‐mattering (Duradoni et al. 2024; Shafiq et al. 2023).

### 4.7 Theoretical Contribution and Implications

From a holistic perspective, the Turkish versions of the four scales demonstrated robust psychometric properties. Across educational and gender groups, the four scales showed coherent factor structures, acceptable reliability, and measurement invariance. Descriptive trends matched the developmental expectations. These demonstrated that Turkish students generally experienced more mattering than anti‐mattering. However, there were slight differences between high school and college students with regard to experiencing the two constructs. Online mattering and anti‐mattering were therefore clearly observed in Turkish students’ experiences. Online social interactions appear to be an important part of how adolescents and young adults experience social inclusion and exclusion. The current results further demonstrate that the adapted measures provide reliable and valid assessments of mattering and anti‐mattering among Turkish adolescents and young adults (Akyazi and Kavas 2025; Demir et al. 2012; Flett et al. 2022; Duradoni et al. 2024).

The results contribute to the existing theory by showing that mattering and anti‐mattering are both psychometrically sound and conceptually relevant in a Turkish cultural context (Flett and Nepon 2024; Duradoni et al. 2024). These results are supported by the consistency of the factor structures and reliability of these structures across developmental groups. This justifies the generalizability of mattering‐related constructs across non‐Western samples and underpins the relevance of the findings in cultural contexts (Akyazi and Kavas 2025; Demir et al. 2012).

The findings also reveal that social significance perceptions were cross‐platform (offline and online). These demonstrated the integrated aspects of digital and face‐to‐face social lives among modern youth (Shafiq et al. 2023; Duradoni et al. 2024). The slightly increased online mattering in high school students might be an indication of developmental and cultural trends of technology‐mediated socialization. The lower anti‐mattering in college students is correlated with greater autonomy and social confidence in emerging adulthood (Arnett 2000; Odgers and Jensen 2020). One possible explanation for this observation is that in a college, Turkish students are probably more independent in their education and can widen their peer circle. This is a general developmental change that is typical in emerging adulthood (Arnett 2000; Odgers and Jensen 2020).

Taken together, these results confirm the presence of theoretical models where mattering and anti‐mattering are differentiated and where cultural and contextual factors are important in predicting social evaluative experiences. The research highlights the need to use culturally modified measurement instruments to promote international investigations in the areas of social meaning and psychological health (Akyazi and Kavas 2025; Flett and Nepon 2024).

### 4.8 Practical and Cultural Implications

The validated scales of mattering and anti‐mattering have practical use in educational and mental health settings working with adolescents and emerging adults. Educational institutions can also integrate these instruments into the screening and assessment procedures to detect students with low perceived social importance or high anti‐mattering and to provide early and specific assistance (Flett and Nepon 2024; Duradoni et al. 2024).

Both offline and online mattering can inform educators to implement interventions that enhance inclusive peer settings and positive digital interaction, including structured peer‐support programs and digital citizenship programs (Shafiq et al. 2023; Flett et al. 2022). Mental health professionals can also use these results to create culturally sensitive programs that can help increase the sense of mattering and diminish the sense of invisibility, especially by using family‐based or group‐oriented programs (Demir et al. 2012; Akyazi and Kavas 2025).

From a cultural perspective, these findings are especially important in Turkey. Here, social relationships with family and peers play a vital role in daily life. As a result, young people's sense of social value and belonging is strongly influenced by these interpersonal connections. The results of the online setting indicate the need to encourage positive online communication and supervision of spaces that may lead to a sense of exclusion. Initiatives and policies that encourage positive online comments and social inclusion can decrease anti‐mattering experiences among youth (Duradoni et al. 2024; Shafiq et al. 2023).

### 4.9 Limitations and Future Directions

There are a few shortcomings in this contribution that must be noted. To begin with, the cross‐sectional design does not allow any causal interpretations of the relationships between mattering, anti‐mattering, and psychosocial outcomes (Arnett 2000; Flett and Nepon 2024). Both longitudinal and experimental designs will be necessary to determine the developmental pathways (Duradoni et al. 2024). Second, the self‐report measures employed in the present contribution were likely to be subject to response biases, including social desirability and common‐method bias (Flett et al. 2022; Shafiq et al. 2023). In the future, multi‐informant designs or ecological momentary assessment strategies might come in handy.

Although the study included two samples (high school and college students), the representation of broader cultural and socioeconomic groups was not reflected. Hence, the generalizability might be limited (Demir et al. 2012; Akyazi and Kavas 2025). These results should be replicated in various cultural settings, and other social variables, including family relationships and digital media usage, should be investigated to offer a more detailed picture of perceived social importance (Duradoni et al. 2024; Shafiq et al. 2023).

## 5 Conclusion

This research offers a thorough psychometric analysis of mattering and anti‐mattering scales, and it has been shown that they are reliable, structurally valid, and measurement‐invariant in Turkish adolescents and emerging adults (Flett and Nepon 2024; Akyazi and Kavas 2025). According to the findings, mattering is typically observed at higher levels than anti‐mattering and both the offline and online dimensions reflect significant meaning of social relevance across different developmental stages (Duradoni et al. 2024; Flett et al. 2022).

The combination of offline and digital environments in a single assessment system enables the study to contribute to the empirical knowledge of how social value and invisibility are experienced by young people in modern settings. Such findings form a solid basis for future studies and can justify the implementation of culturally adjusted mattering scales in research and practice to facilitate social connectedness and psychological well‐being (Demir et al. 2012; Shafiq et al. 2023). Figure 1, 2, 3, 4 Table 4, 5, 6, 7

## Author Contributions

Zane Asher Green: Writing – review and editing, Writing – original draft, validation. Alican Kaya: conceptualization, investigation, Writing – original draft, methodology, Writing – review and editing, data curation, resources. Nuri Türk: Writing – review and editing. Abdulselami Sarıgül: Writing – review and editing. Mehmet Şata: Writing – original draft, Writing – review and editing, formal analysis, visualization. Murat Yıldırım: Writing – original draft, Writing – review and editing, validation, supervision, resources. Mirko Duradoni: Writing – review and editing.

## Funding

The authors have nothing to report.

## Ethics Statement

All procedures performed in studies involving human participants were in accordance with the ethical standards of the institutional and/or national research committee and with the 1964 Helsinki Declaration and its later amendments or comparable ethical standards. The research was approved by Agri Ibrahim Cecen University Ethics Committee (reference number: 135734).

## Consent

Consent was obtained from all the participants included in the study.

## Conflicts of Interest

The authors declare no conflicts of interest.

## Data Availability

The datasets generated and/or analyzed during the current study are available from the corresponding author upon reasonable request.
